# Supplementary material for: The association of depression with subsequent dementia diagnosis: A Swedish nationwide cohort study from 1964 to 2016
Source: PLoS Med. 2020 Jan 9;17(1):e1003016. doi: 10.1371/journal.pmed.1003016 (PMC6952081; doi:10.1371/journal.pmed.1003016)
Supplement: Attachment — Submitted filename: Authors response_final.docx [file pmed.1003016.s002.docx]

**Requests from the editors:**

1. Did your study have a prospective protocol or analysis plan? Please state this (either way) early in the Methods section.

**Authors’ response:** We did not have a prospective protocol for the analysis, however, the analysis that we present analysis were preplanned. We have added a statement about this in the Methods section, p.8. row 189-190.

a) If a prospective analysis plan (from your funding proposal, IRB or other ethics committee submission, study protocol, or other planning document written before analyzing the data) was used in designing the study, please include the relevant prospectively written document with your revised manuscript as a Supporting Information file to be published alongside your study, and cite it in the Methods section. A legend for this file should be included at the end of your manuscript.

**Authors’ response:** Please see above.

b) If no such document exists, please make sure that the Methods section transparently describes when analyses were planned, and when/why any data-driven changes to analyses took place.

**Authors’ response:** In our revision, aligned with the comments from reviewers, we have presented the specific study hypotheses in the Introduction (p.6, row 132-135) revised the Methods section for better transparence.

c) In either case, changes in the analysis—including those made in response to peer review comments—should be identified as such in the Methods section of the paper, with rationale.

**Authors’ response:** Please see above. In addition, we did not change the analysis method in response to review comments.

2. You have noted restrictions apply regarding the availability of your data.  PLOS Medicine requires that the de-identified data underlying the specific results in a published article be made available, without restrictions on access, in a public repository or as Supporting Information at the time of article publication, provided it is legal and ethical to do so. Please see the policy at 

<http://journals.plos.org/plosmedicine/s/data-availability> 

and FAQs at 

<http://journals.plos.org/plosmedicine/s/data-availability#loc-faqs-for-data-policy>  

**Authors’ response:** According to Swedish law we are not allowed to submit data on Swedish citizens to any public repository, regardless of whether the social security number and name of each individual in the file has been removed.

3. Please clearly state somewhere in the main text the primary outcome of the study, and secondary outcomes if applicable (e.g. diagnosis of dementia or death).

**Authors’ response:** We have added in the last paragraph of the Introduction that the primary outcome of the study was dementia diagnosis, p.6, row 129.

4. Title: Please change “risk of” to “association with” in the title, as this is an observational cohort study and we want to avoid any implication of causality.

**Authors’ response:** We appreciate being clear about the observational design and avoid confusions about the study methodology, and have changed the title according to your suggestion to: “Depression and the association with subsequent dementia diagnosis: A Nationwide Cohort Study”

5. Abstract Background: The final sentence should clearly state the study question.

**Authors’ response:** We have added a final sentence to the background section in the abstract stating the study question, p.2, row 31-33.

6. Abstract Methods and Findings: Please define the years during which the study took place.

**Authors’ response:** We have added to the years of the study in Methods and Findings, p.2, row 37.

7. Abstract Methods and Findings: Please quantify the main results comparing dementia diagnoses in those with and without depression, and the sub-analyses, with p values in addition to the 95% Cis.

**Authors’ response:** We have added p values in as requested, p.2, row 44-51.

8. Abstract Methods and Findings: Please include the important dependent variables that are adjusted for in the analyses.

**Authors’ response:** We have included the dependent variables adjusted for the in Abstract Methods and findings, p.3, row 51-52.

9. Abstract Methods and Findings: In the last sentence, please describe the main limitation(s) of the study’s methodology.

**Authors’ response:** We have added the main limitations of the study in the Abstract Methods and Findings, p.3, row 52-53.

10. Methods: Please indicate the form of consent obtained, or the reason that consent was not obtained (e.g. the data were analyzed anonymously).

**Authors’ response:** We have added under Ethical Considerations, p.9, row 216.

11. Methods: For all observational studies, in the manuscript text, please indicate: the specific hypotheses you intended to test, and when reported analyses differ from those that were planned, transparent explanations for differences that affect the reliability of the study's results. If a reported analysis was performed based on an interesting but unanticipated pattern in the data, please be clear that the analysis was data-driven.

**Authors’ response: As presented above,** we have presented the study hypotheses used in the last paragraph of the Introduction. These hypotheses were derived from a recently published study on the association between traumatic head injury and dementia (published in Plos Medicine 2018).

12. Results: Please provide 95% CIs and p values associated with the analysis of differences between cohort demographic factors (data presented in Table 1).

**Authors’ response:** We have provided more detailed information on the results from the Chi-square and T-test used to investigate the differences between depressed (case) and non-depressed groups (control) on demographics and health status at baseline in the Results, p.10, row 227. For further statistics we refer to Table 1.

13. Results: Please provide the p values in addition to the 95% CIs for the odds ratios of depression and risk of subsequent dementia diagnosis in the non-sibling cohort (Table 2 data).

**Authors’ response:** We have provided p values as requested.

14. Results: Please provide the p values in addition to the 95% CIs for the odds ratios of depression and risk of subsequent dementia diagnosis in the sibling cohort (Table 3 data).

**Authors’ response:** We have provided p values as requested.

15. Results: Please provide the p values in addition to the 95% CIs for the odd ratios of the associations between depression severity/type and dementia diagnosis (Table 4 and 5 data).

**Authors’ response:** We have provided p values as requested.

16. Results: For all the adjusted analyses described, please also provide the unadjusted analyses.

**Authors’ response:** We have provided unadjusted analyses for all adjusted analyses as requested (Table 2, Table 3, Table 4, Table 5).

17. Figure 1: Please indicate in the figure caption the meaning of the black and gray lines.  Please display the 95% CIs

**Authors’ response:** The black line represents the hazard ratio and the shaded grey areas the 95% CIs. We have edited the figure and made the black line thinner so that the 95% CIs hopefully are easier to identify. We have also updated the legend to Figure 1 (page 26).

18. Table 1: Please provide statistics to illustrate the factors for which the individuals with and without depression were significantly different, as stated in the Results.

**Authors’ response:** We have provided the results from the Chi-square and T-test used to investigate the differences between depressed (case) and non-depressed groups (control) on demographics and health status at baseline, including 95% CI and p values in Table 1.

19. Table 2 and Table 3: “Confident intervals” should be “confidence intervals” in the Table legend.  Please provide p values along with the 95% CIs for all comparisons.  Please define the abbreviations “OR” and “CI” used in the tables.

**Authors’ response:** We have changed Table legend to “confidence intervals” and provided p values and defined abbreviations as requested.

20. Table 4 and Table 5: For the adjusted analyses described, please also provide the unadjusted analyses.  “Confident intervals” should be “confidence intervals” in the Table legend.  Please define the abbreviations “OR” and “CI” used in the tables.  Please provide p values along with the 95% CIs for all comparisons.

**Authors’ response:** In Table 4 and 5 we have changed Table legend to “confidence intervals” and provided the unadjusted analysis and p values and defined abbreviations as requested.

21. Table 5:  The footnotes denoted by * and ** are repeated.

**Authors’ response:** We have revised the footnotes.

22. Please use the "Vancouver" style for reference formatting, and see our website for other reference guidelines:
<https://journals.plos.org/plosmedicine/s/submission-guidelines#loc-references>

**Authors response:** We have adjusted the reference formatting.

23. Please ensure that the study is reported according to the STROBE guideline (or the most relevant guideline: <http://www.equator-network.org/>), and include the completed STROBE checklist as Supporting Information. When completing the checklist, please use section and paragraph numbers, rather than page numbers. Please add the following statement, or similar, to the Methods: "This study is reported as per the Strengthening the Reporting of Observational Studies in Epidemiology (STROBE) guideline (S1 Checklist)."

**Authors response:** We have ensured that the study is reported in coherence with the STROBE guidelines and added a statement on this, p.6, row 139-140. Please see attached STROBE checklist submitted as Supporting information.

**Comments from the reviewers:**

Reviewer #1: The work uses routinely-collected data sources (EHR records from a country with UHI) to ascertain timing and incidence of dementia and depression, and then estimates the differences in risk of a record of dementia according to a previous diagnosis of depression. My role in the review process is as a statistical reviewer, and I have made some comments on the interpretation of the results with regard to the data sources as well. I can appreciate the work that has gone into this study, creating matched cohorts of this size from EHR data poses many challenges.

Statistical Issues:
The first statistical issue comes from the comparison of odds ratios between the general population cohort and the sibling-matched cohort. The incidence of the key outcome is different in the two populations (9,802/238,772 in the pop cohort, vs 1,161/50,644 in the sibling cohort). The odds ratio is not collapsible, so that a similar odds ratio between the two cohorts at the same time after diagnosis may actually reflect dissimilar relative risk (or a dissimilar odds ratio, and a similar relative risk). This issue affects comparison of ORs over time, and between single vs. multiple episodes, men vs. women etc. Estimating a rate ratio (estimated by log-binomial or log-Poisson with robust SE) is a way to avoid this issue, as is estimating relative risk from marginal estimates of risk (e.g. predictive margins in Stata).

**Authors’ response:** We appreciate the thorough review provided in relation to the statistical methods. Regarding the first statistical issue proposed by the reviewer, we find the point made by the reviewer about potential problems related to collapsibility of odds ratios when analyzing different subgroups highly relevant. A problem may occur where the relative risk in two groups are similar, still, the corresponding odds ratios are dissimilar. The reviewer suggests that our comparisons of odds ratios across groups is problematic because the relative risks may be different despite similar odds ratios (or vice versa). However, the odds ratios approximate the odds ratios when the outcome incidence is low (< 10%) and the problem with non-collapsibility decreases with lower incidence (Rothman, Greenland, & Lash, 2008). The incidence of dementia was relatively low in our study, so the odds ratios approximate relative risks. For example, the crude odds ratio comparing depressed and non-depressed subjects in the large cohort is 2.21 (=[5.66%/(100%-5.66%)]/[2.55%/(100%-2.55%)]) and the risk ratio is 2.12 (=5.66%/2.55%); in the sibling cohort, the crude odds ratio is 2.98 (=[3.41%/(100%-3.41%)]/[1.17%/(100%-1.17%)]) and the relative risk is 2.91 (=3.41%/1.17%). The same applies for the estimated odds ratios over time. For example, in the large cohort where the crude odds ratio comparing depressed and non-depressed subjects approximates relative risk over the different time intervals of time after depression diagnosis, e.g. in the time interval of 0-5.9 months after depression, crude odds ratio of 13.79 and risk ratio is 13.69 and in the time interval of 10-20 years after depression diagnosis crude odds ratio is 1.43 and risk ratio is 1.42 (see Table 2). These similarities indicate that our use of odds ratios is not problematic, so we see little need for changing the regression models. To better illustrate this, we have added incidence of dementia (number of individuals and percent) separately for case and control for the different time intervals in Table 2 and in Table 3.

The second statistical issue relates to the selection of covariates. What criteria was used for the selection of comorbidities subsequently used as adjustments for confounding? P-values for univariable association or effect size? Univariable pre-filtering can have unintended consequences - there is a good review by Heinz and Dunkler (Transplant International 2017; 30: 6-10) on this subject.

**Authors’ response:** The covariates were selected based on previous known associations with the exposure, i.e. depression or the outcome of dementia. Thus, the covariates included also risk factors for cardiovascular disease, as these are important risk factors for vascular dementia, as presented in page 8, line 179-183.

Data sources:
The foundation of this work is the quality of the data collection used to identify the outcome, exposure, and the potential confounders. Is there a key reference that about the timing and composition of data collection in the SNPR? It looks like it is composed of hospital and specialist care - is there general practice (family practice) collection included? Many of the PLoS Medicine readers won't be familiar with the health system behind the data collection and may need some context to understand what is accomplished in this work. Was there missing data in the original data collection that precluded matching? How many records couldn't be used if so?

**Authors’ response:** The key reference Ludvigsson et al. (2011) is an external review and validation study of the SNPR and provides detailed information on the history, structure, coverage and quality of the SNPR. We have described the SNPR in greater detailed under Procedure, p 7, from row 169. Data from general practice is not included into SNPR due to the lower quality of data. Thus, some of the controls could have been diagnosed with depression and both controls and cases could have been diagnosed with dementia in primary care. This would, if anything, attenuate the associations, and most likely in a similar manner during the follow up time. The total number of cases that was excluded due to missing data, or depression, or dementia diagnoses made post mortem precluding the matching procedure was low very low in comparison to the total cohort considered for inclusion (n=813, <0.001%). We have provided information on missing in the original data register (SNPR) in Material and Methods, p. 6, row 145.

There is good evidence overall the SIR data accurately captures many conditions (from Ludvigsson et al 2011) and that previous validation studies have shown that a general dementia diagnosis is captured accurately.  How well is depression exposure captured if the records are from acute (hospitalised) and specialist care episodes? In my country the majority of depression is treated in general practice (family practitioner) or by clinical psychologists and only severe cases of depression would be treated as an in-patient or by a psychiatrist. Is this a similar situation to Sweden? Are there any validation studies for PPV and sensitivity of timing of depression from the SNPR for depression of differing severity?

**Authors’ response:** These are very insightful questions by the reviewer, although difficult to address. In general most cases of depression is likely treated in primary care, as in the reviewers country, and also more severe cases are probably more often treated in specialist care. We are also not aware of any study that has evaluated PPV and sensitivity of timing of depression from the SNPR for depression of differing severity. Yet, despite these potential shortcomings, in the present study individuals diagnosed with a severe depression had an increased risk of dementia more than 20 years compared to less than 10 years for a diagnosed mild depression.

There is clearly a time-dependence, and the odds of dementia diagnosis are very high immediately following a diagnosis of depression. This is hinted at in the discussion but I think needs to be discussed clearly - could the depression actually an unrecognised sign or symptom of early dementia (potentially reversing the causal mechanism) or is it due to confounding according to more intense health service use increasing the diagnosis of both diseases?

**Authors’ response:** The reviewer makes a good point about the potential explanations of the increased odds of dementia with short follow-up from depression diagnosis. We have made changes to ensure we discuss this clearly in relation to limitations of our study on p. 14, row 331-334.

Are there any known confounders of the depression-dementia relationship unavailable in the SNPR data? What level of confounding would be needed to account for the observed relationship? I have found the E-value is a useful tool (Ann Intern Med. 2017;167(4):268-274.) to consider how sensitive an observed outcome-exposure is to an unmeasured confounder.

**Authors’ response:** Again a very insightful comment, although impossible to answer for a number of reasons. First, we need to understand the measurement errors influencing the diagnosis of dementia and depression, and the different types of dementia, and the different severity of depression (as demonstrated the association was stronger for severe depression and the outcome of vascular dementia). These measurement errors would attenuate the associations found. Secondly, we need to know the measurement errors influencing the different covariates used in the present study. Third, we need to know whether the covariates used are confounders or mediators, that perhaps should not be included in the analyses. Finally, we likely miss some covariates, although we have been able to include the most important, except perhaps for cognitive function in young adulthood. Finally, and perhaps most importantly, adjustment for all covariates changed the associations minimally, and most hazards ratios actually increased slightly.

Specific comments:
p2, Conclusions. Logistic regression is used throughout this manuscript, so 'increased odds' of dementia is a more accurate description of the results.

**Authors’ response**: We have changed “increased risk” to “increased odds” at most places in the paper.

p2, Conclusions. The phrasing part of this sentence 'of especially vascular dementia' is unclear. I would either just refer to dementia generally or reword this sentence to include the additional information about vascular dementia.

**Authors’ response**: We have changed this sentence to refer to dementia in general.

P15, Household income. Is there a way to indicate what the relative difference in household income is for international readers (i.e. converted to USD/Euros or an indication of household income distribution in Sweden at the time of the data collection)? The difference in household income between those diagnosed with depression and not diagnosed with depression in the sibling cohort looks extreme - but this could just be my unfamiliarity with the currency.

**Authors’ response:** To better illustrate the relative difference in household income for international readers, we have provided the household income in USD in addition to Swedish krona, Table 1. We discovered a typing error in the household income variable in the sibling cohort, which indicated an extreme difference between depressed and non-depressed subjects, this has now been corrected.

P16, Title of table. Although it is possible to identify the source of the data from the reported N, it would be easier if the source of the data (e.g. Population matched cohort) was also included as in Table 3.

**Authors’ response:** Good point! We have provided a title for Table 2 & 3.

P19, Table 5. There is a 'ns' indicator for single depressive episode and 10-20 years after diagnosis, but this doesn't appear elsewhere in the table where the 95% CI overlaps with 1. I would suggest to just remove the 'ns' as the CI already gives this information.

**Authors’ response:** We have removed the “ns” in Table 5. In addition, we have provided p values in response to editors’ requests.

**Reviewer #2:** PMEDICINE-D-19-03130 "Depression and the risk of subsequent dementia diagnosis: A Nationwide Cohort Study" 

In this study the authors examined the association between depression and dementia and reported depression to be associated with "an increased risk of especially vascular dementia, even more than 20 years after the depression, and the association remained after
adjustment for familiar factors."
The data in the study come from two cohorts formed of all individuals above 50 years, living in Sweden. The first cohort was composed of individuals diagnosed with depression matched to controls without depression (n=238,772). The second was a co-sibling cohort (n=50,644) consisting of same-sex full sibling pairs with discordant depression status. This is an impressive study design, and the size of the study sample would lead us to believe that the results would be robust. Nevertheless, the following points need further reflection.

1.Novelty
The authors say
"The association between depression and subsequent dementia diagnosis has been established
in at least four independent meta-analysis indicating close to a twofold risk of developing
dementia followed by depression [7-10], as well as several reviews [11-14]."
Given the meta-analyses and reviews on this topic, the contribution of this study is unclear.

**Authors’ response:** The novelty of our study mainly lies in the long follow up of over 20 years and the large nationwide cohort, in combination with adjustment for familial factors by the use of a co-sibling design. To the best of our knowledge, this has not been done previously. To better emphasized this, we have adjusted the section following the citation by the reviewer, p. 5, row 112 and onward.

2.Depression diagnosis
Diagnosis of depression came from the Swedish National Patient Register. The manuscript provides the ICD codes for diagnosis but no detail on the register itself. Further information is required on the sources of data used in this register, in terms of consultations - family doctor, psychiatrists, hospitals? Is the diagnosis based on prescription of antidepressants or hospitalization for depression? This information is needed to understand what is being measured by the ICD codes. The target population was 3.3M and only 119,386 cases of depression were identified - this is a surprisingly low rate.

**Authors’ response:** This is an insightful comment by the reviewer. In response we have described the SNPR in greater detailed under Procedure, p.7, row 167 and onward. The key reference Ludvigsson et al. (2011) is an external review and validation study of the SNPR and provides detailed information on the history, structure, coverage and quality of the SNPR. With respect to the low rate of depression, only diagnoses set in specialist care are included into SNPR, because diagnoses set in primary care in general have to low quality. Thus, some cases of both depression and dementia will be missed, which may attenuate the association between these diagnoses.

3.Matched controls 
In the first cohort, controls were matched for age, sex, and citizenship but Table 1 shows cases and controls to differ systematically on sociodemographic and health status. It is possible that the results can be explained by systematic differences in these two groups that have not much to do with depression.

**Authors’ response:** The reviewer has correctly noted that the depressed and non-depressed groups systematically differ on socioeconomic factors and health status at baseline and that this may influence the found association between depression and subsequent dementia. Therefore, we adjusted the regression models for both socioeconomic factors and health status at baseline, and have provided the unadjusted and adjusted odds ratios for all analyses. Hence, we find it unlikely that the found association is largely influenced by confounding. It should also be noted that the adjusted ORs differed minimally from the unadjusted, any many of them actually increased slightly. Still, potential confounding beyond the factors we were able to control for in our study is important to address, which we discuss as a potential limitation of our study on p. 14, row 332: “… even though controlling for civil status and household income, we were not able to account for additional socioeconomic factors, e.g. education level.”.

4.Dementia diagnosis
Further details are required on how dementia was diagnosed in both cohorts. Do the data on dementia come primarily from mortality registers? Both cohorts in the manuscript have a follow-up of over 20 years but the rate of dementia is 4.1% and 2.3%. This is again surprising low. Is it possible that most cases of dementia were missed in the register?

**Authors’ response:** As explained above, SNPR only registers diagnoses set in specialist care with resulting relative low risk rate.

5.Conclusion
I do not think that the conclusion "The results of the present nationwide study indicated an increased risk for dementia diagnosis after depression, even when the depression occurred 20 years or more before the dementia diagnosis" is supported by the data presented in the manuscript. Figure 1 clearly shows reverse causation (or depression being a prodromal feature of dementia) as the excess risk drops at Year 5 of follow-up and remains unchanged thereafter. There are problems with measurement of exposure and the outcome, and the selection of the control group. Furthermore, the analysis of the 20-year follow-up is based on a small number of cases (481 cases in 26,044 persons in cohort 1, and 76 cases in 6,296 persons in cohort 2), making it difficult to draw conclusions.

**Authors’ response:** Throughout the paper we have made changes to refer to increased odds instead of increased risk to avoid any indication of the causality of the found. As can be seen in Figure 1, as well in Table 2 and 3, the association was strongest initially and then decreased rapidly, however remained significant over a follow-up of more than 20 years (see Table 2 & Table 3). Although the analysis in the interval after 20-year follow-up is based on a rather small number of cases in the sibling cohort, the results are consistent with the findings in individuals with shorter follow up time, and statistically significant both in the smaller sibling cohort and the larger matched cohort, which we find sufficient to draw the conclusions presented. We are not sure what the reviewer refers to when writing that there are problems with measurement of exposure and the outcome, and the selection of the control group. Regardless, we hope that the reviewer will agree that any measurement errors affecting the exposure and outcome will if anything attenuate the associations found.

**Reviewer #3:** This study on risk of dementia among older depressed adults should be of substantial interest to readers of the journal.

In the Introduction, the authors note the high prevalence of dementia and the need to identify modifiable dementia risk factors.  They briefly cite literature noting the increased risk of dementia among those with depression, noting that depression may be seen as a discrete risk factor or as a dementia prodrome.  

In the final introductory paragraph, the authors note that the purpose of the study was to investigate if the association between depression and later dementia risk is time dependent, identify factors that influence the association, and whether the association is influenced by severity and frequency of depression.  Given their prior literature review in the preceding paragraphs, one wonders whether the authors have any a priori hypotheses in these three areas, or if this is truly a completely exploratory study, which would require appropriate statistical correction.  If the authors did have a priori hypotheses, they should state them.

**Authors’ response:** We have stated the specific study hypotheses used in the study (p.6, row 130-133), which were based on a previous recently published study where we found a strong association between traumatic head injury and dementia in a similar nationwide cohort with the same experimental set up as in the present study (Nordström A PLoS Medicine 2018).

In the Materials and Methods section, the authors describe reasonably well how the cohorts for the present study were derived.  They refer to “depression” as opposed to “major depression.”  If indeed they are focusing on major depression, they should be more precise.  This is also the case for the next section, “Procedure,” in which they specify ICD-10 codes of F32 and F33 but only mention “diagnosis of depression.”

**Authors’ response**: The reviewer has correctly noted that our study focuses on Major depression, and we have made changes accordingly in the Materials and Methods section and the Procedure section, p.7, row 168 and onward.

They note that “all diagnoses were set in specialist care.”  It is not clear what that means.

**Authors’ response:** We understand the confusion and have removed this notation in the discussion, as well as provided a more thorough explanation of the data sources for the diagnoses used in our study under Procedure, page 8, row 181 and onward.

In the statistical methods section, the authors quickly jump to time dependency analyses without first indicating what initial analysis, if any, that they undertook comparing depression and dementia risk.  They should specify how the dependent and independent variables were constructed.  They should justify why separate analyses were performed for men and women, as opposed to, for example, doing separate analyses for different age cohorts, given differences in typical ages of onset for Alzheimer’s disease and vascular dementia.

**Authors’ response:** In our revisions, we have presented the study hypotheses used in our study, which we believe will give a better understanding of the analysis methods chosen p.6, row 130-133 which were based on a previous recently published study where we found a strong association between traumatic head injury and dementia in a similar nationwide cohort (Nordström A PLoS Medicine 2018). We have made changes in the introduction to better motivate why we performed separate analysis for men and women (p.5, row 123-124). Investigating the association between depression and dementia for different age cohort may further illustrate the nature of the association, based on the differences in typical onset of Alzheimer’s disease and vascular dementia. However, we believe that the differences in the association based on type of dementia is better described by separate analysis performed for Alzheimer’s disease and vascular dementia as we present in our study, rather than analyses performed on different age cohorts.

It is not clear what the following sentence means: The knots were set in default positions.

**Authors’ response:** This is a statistical term to describe where the curves constructed using the parametric flexic models were allowed to bend.

If this is an exploratory study without hypotheses, then it is not clear that appropriate statistical corrections were employed.

**Authors’ response:** The study was not truly exploratory and we have provided the specific hypotheses tested, as stated above.

The results, in the text, tables and figures, are clearly presented. 

In the Discussion, the authors summarize some of the main findings, but should mention their findings related to severity as well.

**Authors’ response:** In the discussion we discuss our finding related to severity of the depression episodes and have added the results regarding frequency of depressive episodes as well, p. 13, row 320-321.

They then place their study in the context of prior literature.  There are a few key studies that they should consider including:

Barnes et al. Arch Gen Psychiatry. 2006;63(3):273-279.  Study focusing on depression as risk for MCI

Steffens et al.  Biol Psychiatry. 1997;41(8):851-856.  Large epidemiological study noting depression as risk for AD only when there is a few years between depression onset and dementia onset

*Kokmen et al.  Neurology. 1991;41(9):1393-1397.  Large population study linking episodic depression and AD.*

Becker  et al.   Am J Geriatr Psychiatry. 2009;17(8):653-663. A community based study that found that depressed mood is not a risk factor for incident dementia.

Steffens et al.  Am J Geriatr Psychiatry. 2007 Oct;15(10):839-49.  Clinical study noting depression as risk for non-AD dementias.

Inclusion of some of these and other studies might allow for a fuller discussion of their findings.

**Authors’ response:** We appreciate the recommendation regarding key studies that could increase the quality of the discussion section. We have included some of these studies and find them highly relevant to discuss in relation to our results.

The authors identify appropriate limitations in their study.  If this truly was an exploratory study (without a priori hypotheses), that might also be considered a limitation.

**Authors’ response:** We agree! We have provided the specific hypotheses used in our study, as explained above.

**References:**

Ludvigsson, J. F., Andersson, E., Ekbom, A., Feychting, M., Kim, J. L., Reuterwall, C., . . . Olausson, P. O. (2011). External review and validation of the Swedish national inpatient register. *BMC Public Health, 11*, 450. doi:10.1186/1471-2458-11-450

Rothman, K., Greenland, S., & Lash, T. (2008). *Modern epidemiology: Third edition*. Philadelphia, PA: Lippincott Williams & Wilkins.
