# Supplementary material for: The association of depression with subsequent dementia diagnosis: A Swedish nationwide cohort study from 1964 to 2016
Source: PLoS Med. 2020 Jan 9;17(1):e1003016. doi: 10.1371/journal.pmed.1003016 (PMC6952081; doi:10.1371/journal.pmed.1003016)
Supplement: Attachment — Submitted filename: Authors_response_191210.docx [file pmed.1003016.s003.docx]

**Authors’ response**

1. Data Availability Statement: Thank you for your previous response that “Data from the Swedish National Patient Register (SNPR) can be requested from the National Board of Health and Welfare in Sweden (www.socialstyrelsen.se). The background data used in the study can be requested from Statistics Sweden (www.scb.se).” Please update the data availability statement on the manuscript submission form. Please also include whether there is/ whether you paid a fee to access the Swedish National Patient Register data.

**Authors’ response: Done**

2. Response to reviewer 1: Thank you for your thoughtful response regarding the discussion of odds vs. risk ratios, and I appreciate your opinions on this. However, as requested by the reviewer, please include at least a brief discussion of the use of odds ratios rather than risk ratios as a limitation.

**Authors’ response: We have in response added the following (line 185): “A potential limitation in the present study is the use of odds ratios instead of risk ratios, since the use of odds ratios can result in non-collapsibility, i.e. that estimated odds ratios are not similar to the relative risks. However, this potential problem seems to be of minor importance as the estimated relative risks and odds ratios for the different time intervals were similar in the present study.”**

3. Discussion: Line 179 and response to reviewer 2: Please elaborate on your sentence: “This may also explain the lower than expected rates of depression and dementia.” For example, what are the expected rates? And please elaborate slightly on how this explains the low rates.

**Authors’ response: We have in response added the following: “This may also explain the lower than expected rates of depression and dementia in our study, since many patients are diagnosed exclusively in primary care. Thus, previous studies have indicated the prevalence of both depression and dementia is around 10% in older community dwelling citizens.”**

4. Discussion: Line 178: I understand your meaning here, but please clarify the sentence ”...found association may not be generalizable for diagnoses of depression set in primary care.” to make it clear that you are saying that a limitation of your study is that the results here may not necessarily generalize to individuals who were diagnosed in a primary care setting because the individuals included in the study were diagnosed in specialty care, and as such may represent more severe cases of depression/dementia.

**Authors’ response: In response we have added the following (line 175): “Additionally, a limitation of our study is that the results here may not necessarily generalize to individuals who were diagnosed in a primary care setting because the individuals included in the study were diagnosed in specialty care, and as such may represent more severe cases of depression/dementia.”**
